# Clinical and surgical physician’s perception of nutrition knowledge

**DOI:** 10.1186/s12875-024-02534-x

**Published:** 2024-08-03

**Authors:** María Belén Ocampo-Ordóñez, Ivonne Headley, Emily Sofía Arévalo-Alvear, Heather Wasser, Andrea Carolina Román-Sánchez

**Affiliations:** 1 Escuela de Salud Pública y Nutrición, Universidad San Francisco de Quito, Diego de Robles y Pampite, S/N, Quito, Quito, Ecuador; 2 Gillings School of Global Public Health, University of North Carolina at Chapel Hill, Chapel Hill-EE.UU, NC USA

**Keywords:** Nutrition care, Physicians, Attitudes, Beliefs

## Abstract

**Background:**

Due to the significant increase in the prevalence of food-related diseases, the value that physicians place on nutritional advice may have implications for patient treatment. The objective of this study was to evaluate the perception of the importance of nutritional intervention among physicians in the Universidad San Francisco de Quito’s (USFQ) healthcare system.

**Methods:**

This cross-sectional study employed a telephone survey administered to a subset of all medical doctors (MDs) working in the healthcare system clinics of USFQ between 2021 and 2022. Study participants were recruited through voluntary response sample from a complete list of 253 MD. The single time questionnaire consisted of a 22-item validated survey in which attitudes, self-perceived capacity, and knowledge about nutrition ofmedical doctors were evaluated. Data was analyzed using descriptive statistics, two-sided t test, bivariate associations and linear and logistic regressions.

**Results:**

136 MDs completed the survey yielding a response rate of 54%. Our analysis grouped participants into clinical (CE) and non-clinical specialties, hereafter referred to as surgical MDs. While a higher percentage of physicians in CE are confident in their ability to provide examples of recommended food portions based on national or international guidelines, 1 in 10 do not know how to use and interpret BMI or waist circumference, and around 1 in 3 do not know how many calories there are in one gram of fat, protein, or carbohydrates, and their basic metabolic functions. Almost all survey participants believe MDs can have an impact on the eating behavior of a patient if time is used to discuss the problem, however, almost half of survey participants believe nutrition counseling is not an effective use of time.

**Conclusion:**

It is important to explore the perceptions and self-confidence of physicians around nutrition related issues. Our results demonstrated that nearly 1 in 4 surgical MDs do not feel capable of recognizing nutritional risk in patients, which highlights the essentiality of physicians having an updated understanding of basic nutrition principles. Future research should examine how commonly MDs refer patients to nutritionists/dietitians, as well as strategies for improving physician knowledge on basic nutrition concepts.

## Introduction

Nutrition related health problems are a world-wide phenomenon, and Ecuador is no exception [1]. The dual burden of nutrition, that is, non-simultaneous co-occurrence of infectious disease, cardiometabolic disease, overnutrition, and undernutrition [2] can be noted throughout Ecuador and its diverse regions. For example in Galápagos, perceived barriers to healthy diets such as price have been associated with an increased consumption of processed and ultra-processed foods [3], while at the same time, other studies have highlighted the impact of child stunting in the same region [4]. Furthermore, diet-related diseases are not merely limited to obesity [5] There is also high incidence and prevalence of chronic non-communicable diseases such as diabetes and childhood malnourishment, where 1 in 3 children suffers malnutrition [6]. In 2021, diabetes was the second leading cause of death in Ecuador, excluding COVID deaths [7], and 20% of children under 2 years are currently affected by stunting [8]. This is higher than the average for childhood stunting in Latin America and the Caribbean of 11.3% [9] and Ecuador continues to have the highest rate of chronic childhood malnutrition in Latin America after Guatemala [8]. The Ecuadorian health situation occurs during complex geopolitical and socioeconomic conditions related to slowing of the economy, increase in insecurity caused by organized crime, disruptions in oil production, climate-related events, and political uncertainty [10].

Nutrition and dietetics is responsible for the prevention, diagnosis and management of acute and chronic conditions caused by a lack or excess of energy [11]. Due to this considerable increase in urgent health concerns, the importance of health professionals having adequate training and knowledge in nutrition is crucial. Because medical attention usually is the first and most frequent point of contact with a patient, the physician holds a key role in obtaining better results. Nonetheless, most MDs are only able to identify nutritional issues, provide general lifestyle change recommendations and refer the patient to a nutrition professional for further treatment, while nutrition professionals are licensed to provide medical nutritional therapy to patients ( [12]. Additionally, the current organization of the Ecuadorian health care system does not include nutrition services in every level of care, positioning physicians at the frontline for preventing and treating non-communicable diseases and undernutrition [13]. Moreover, while nutritional services are covered by public and private insurance, there is a lack of available professionals to address all nutritional needs [14].

Although physicians recognize that nutrition plays a critical role in health and agree that providing nutritional advice is part of their job, they do not always have the capacity to translate this priority into practice. Based on publicly available information regarding medical program curriculums in Ecuador, it can be inferred that most physicians receive about one nutrition course throughout 5–6 years of medical training. Consequently, nutritional advice is not provided by physicians and there are no significant changes in patient health as it relates to nutrition [11]. Research shows that doctors do not have the nutrition counseling skills to support their patients with basic dietary care, even though it has been shown that timely nutritional intervention reduces morbidity, mortality, illness, and medical costs [15]. Nonetheless, there is a gap in knowledge regarding how lack of nutrition counseling skills among physicians in Ecuador, specifically, impacts patient care.

Due to the considerable increase in the prevalence of nutrition related health conditions and the critical role doctor’s hold in providing nutrition advice to patients, the objective of this study was to evaluate the perception of the importance of nutritional intervention among medical doctors (MDs) in the Universidad San Francisco de Quito´s (USFQ) healthcare system. Our survey included doctor’s beliefs around the value of nutrition as part of medical treatment, their self-confidence in providing nutritional information, their personal knowledge about nutrition, and the time they allocate to address nutrition topics in practice.

## Methodology

### Study design

This prospective cross-sectional study employed a telephone survey administered to a subset of all MDs working in 4 of USFQ’s healthcare system clinics in the city of Quito between 2021 and 2022. USFQ has a fifth healthcare system clinic located in the city of Manta which was excluded for this study due to geographic differences. Participants were selected through a non-probabilistic sample design, according to a complete list of 253 MDs working during the period of 2021–2022. All participants had to sign a consent form before answering the survey and the ethics committee of USFQ reviewed and approved this study (COD 2019-003IN).

The survey was conducted by phone, from November 2021 to April 2022. Data was collected in an online survey repository using Open Data Kit (ODK) survey technology. The response rate was around 54% with a collected total of 136 valid responses. A survey response was considered valid if it was answered in its entirety, that is, zero responses were omitted. The questionnaire was created based on the one used by Vetter et al. in their study ¨What do Resident Physicians Know About Nutrition? An Evaluation of Attitudes, Self-Perceived Proficiency and Knowledge.¨ [16] Vetter et al. developed their survey systematically and with attitude scales, which increased the probability that the resulting measures produced useful and reliable data, demonstrating their validity. The single time questionnaire used in our study consisted of a 22-item validated survey measuring perception about the importance of nutritional treatment among MDs as part of their medical practice, attitudes, and knowledge about certain nutrition concepts as well as their self-perceived capacity to provide nutrition counsel. A pilot sample with similar characteristics to that of the study sample was used to conduct an initial test of the questionnaire. While the pilot survey successfully indicated that no structural changes needed to be made to the survey, reminders were implemented for study sample participants in order to achieve a have greater response rate to the survey.

### Measures

The survey included items designed to elicit MD perceptions about nutrition, specifically: (1) beliefs around the value of nutrition as part of medical treatment, (2) self-confidence in providing nutritional information and in patients to apply information, (3) personal knowledge about nutrition, and (4) time allocated to address nutrition topics in practice. Five items evaluated beliefs, e.g. “*It is important to address the importance of the diet every time a patient is attended*,*”* six items assessed confidence in applying nutrition related concepts, e.g. “*I feel comfortable with my capacity to talk about prevention strategies and nutrition treatment of illnesses such as osteoporosis*,* metabolic syndrome*,* hypertension*,* hyperthyroidism*,* stunting*,* gestational diabetes*,* and others*,” five items assessed knowledge of nutrition topics, e.g. “*I know how to use and interpret BMI and waist circumference*,” and three items assessed beliefs around time allocation during a typical patient appointment, e.g. “*It is NOT worth it to dedicate my time to advise patients with deficient eating patterns about nutrition*.” Each item was assessed using a five-item likert-type scale utilizing the following options: 1 *strongly disagree*, 2 *disagree*, 3 *I am not sure*, 4 *agree*, and 5 *strongly agree*.

Participants self-reported their medical specialty, and this data was used to retrospectively classify participants into two groups: clinical and surgical specialties. We conducted this binary classification because the distinction between the two groups was a notable one. The key difference between the clinical and surgical groups was whether or not time was allocated to address nutritional status. In clinical specialties, an intake of the patient’s nutritional history is typically incorporated into routine medical care. These specialties included MDs who self-identified as cardiologist and nephrologists, among others. On the other hand, surgical specialties encompassed surgical practice areas where the focus of practice lied mainly in successfully executing a medical procedure. These specialties included MDs who self-identified as general surgeons, traumatologist, and plastic surgeons, among others. A complete list of specialties represented in our sample can be found in Table 1.

**Table 1:** Specialties represented within sample

Clinical		Surgical
General Medicine	Dermatology	General Surgery
Gynecology	Pneumonology	Otolaryngology
Pediatrics	Neurology	Plastic Surgery
Internal Medicine	Hematology/Oncology	Cardiovascular Surgery
Family Medicine	Occupational Medicine	Mastology
Endocrinology Nephrology	Rheumatology	Vascular Surgery
Gastroenterology	Urology	Cardiothoracic Surgery
Cardiology	Allergists	Ophthalmology (Optometrist)
Oncology	Sports Medicine	Coloproctology
Audiology		Pediatric Surgery
		Anesthesiology

Demographic and sample characteristic data included self-reported biological sex, age, year of undergraduate and postgraduate graduation, the educational institution where undergraduate studies were completed (considering not all survey participants needed agraduate degree to practice), and whether participants considered themselves a private or public sector MDs. It should be noted that in Ecuador, it is commonplace for MDs to work at more than one location. Thus, while USFQ’s healthcare system belongs to the private sector, therefore, all survey participants worked in the private sector, we also wanted to explore if MDs working for USFQ considered themselves to be a public sector MD due to roles played outside of USFQ’s private healthcare system. We then further categorized educational institutions into public and private universities.

## Analysis

All statistical analyses were performed using Stata - version 18. Because only valid survey responses were used in our analysis, that is, questionnaires that had been fully answered, missing data as it relates to the survey items assessing attitudes and perceptions did not need to be coded. The only missing data encountered in this analysis consisted of miscellaneous age entries and year of graduation. Descriptive statistics were used to assess distributions for all survey items. MDs perception items were run as both continuous and categorical variables. To create the dichotomous, categorical variables, each item was coded as *agree* or *disagree*. Agree included the response options “*strongly agree*” and “*agree*,” while disagree included “*I am not sure*,” “*disagree*,” and “*strongly disagree.*” Bivariate associations between sample characteristics and type of practice (clinical vs. non-clinical) were examined using two-sided t-tests for continuous variables and chi-square tests for categorical variables. Linear regression models with continuous MDs perception items as the dependent variable and type of clinical practice as the independent variable were run to examine differences by type of practice, controlling for sample characteristics found significant in bivariate analysis. A similar series of logistic regression models were run with dichotomous MDs perception items as the dependent variable. Significance was set at *P* < 0.05.

## Results

Sample characteristics by type of clinical practice are presented in Table 1. One hundred and thirty six MDs completed the survey, yielding a response rate of 54%. Slightly more than half (55.2%) of the respondents were female MDs and the majority of the sample worked in the private sector (69.9%). Survey participants represented 19 different undergraduate institutions, 73.7% of which were public universities. Biological sex was the only sample characteristic significantly associated with type of practice, with more females in clinical practice as compared to males.

Associations between MDs perception items and clinical practice are presented in Table 2. All models were adjusted for biological sex.

**Table 2:** Sample characteristics and bivariate associations with type of practice

	Total (*n* = 136)	Clinical (*n* = 85)	Surgical (*n* = 49)
	Mean *±* SD **or** n (%)	Mean *±* SD **or** n (%)	Mean *±* SD **or** n (%)
Female Male	75 (55.2) 61 (44.9)	55 (64.7)** 30 (35.3)	19 (38.8) 30 (61.2)
Years since Undergraduate Degree	18.4 *±* 8.0	18.5 *±* 8.3	18.5 *±* 7.5
Years since Postgraduate Degree	11.7 *±* 7.5	11.8 *±* 7.8	11.7 *±* 7.1
Age	43.1 *±* 7.9	43.3 *±* 8.5	43.3 *±* 6.6
Public Education Private Education	96 (71.1) 39 (28.9)	65 (77.38) 19 (26.62)	31 (63.27) 18 (36.73)
Public Sector Private Sector	41 (30.2) 95 (69.9)	23 (27.06) 62 (72.94)	18 (36.73) 31 (63.27)

* *p* < 0.05, ** *p* < 0.01, *** *p* < 0.001

### Beliefs about the importance of nutrition

On average, 97.7% of clinical physicians and 87.7% of surgical physicians agree it is important to address diet every time a patient is attended to. Those in clinical practice are more likely to be in agreement with the importance of advising patients about the use of supplements and when they are contraindicated compared to those in surgical fields.

### Self-confidence in providing nutrition information

55.3% of physicians in clinical specialties are confident in their ability to provide examples of recommended food portions based on national or international guidelines, compared to less than half of those in surgical specialties. Moreover, across both specialties, at least 3 in 4 physicians believe patients are not motivated to change unless they are sick.

### Personal knowledge about nutrition

1 in 10 clinical physicians do not know how to use and interpret BMI or waist circumference. Even more, around 1 in 3 clinical MDs do not know how many calories there are in one gram of fat, protein, or carbohydrates, and their basic metabolic functions.

### Time allocated to address nutrition topics in practice

Although almost all survey participants believe MDs can have an impact on the eating behavior of a patient if time is used to discuss the problem, almost 10% of clinical MDs and 33% of surgical MDs believe nutrition counseling is not an effective use of time. Those in clinical practice, however, were less likely to agree that dedicating time to provide nutrition advice to patients with deficient eating patterns is *not* a good use of time.

## Discussion

There are 84,705 public workers, including administrative and health staff, registered by the Ministry of Public Health of Ecuador [17]. According to the Instituto Nacional de Estadísticas y Censos (INEC), Ecuador has 40,230 doctors and an average of 23.30 for every 10,000 people [18]. This ratio is in line with the WHO’s recommendation of an average of 23 doctors for every 10,000 inhabitants [19]. Furthermore, these numbers indicate that outside of nutritionists, there are enough physicians in Ecuador to provide basic nutritional advice, compared to other health professions. For example, while nurses can also provide basic nutrition counseling, Ecuador only has 15 for every 10,000 people [17]. This positions doctors as an instrumental key towards preventing, diagnosing and implementing basic nutritional recommendations among patients. In fact, the majority of patients consider that the doctor is the most suitable interlocutor for nutritional problems or doubts regarding food [20].

Table 3 summarizes findings as separated by clinical and surgical specialties because of the different nutritional approaches between these practices, more specifically time allocation during consultation for both categories. In clinical specialties, the patient’s nutritional history is considered part of their medical interview. Contrary, surgical specialties focus on the procedure itself, whether or not nutritional status is accounted for. First, there was a significant difference between 97.7% of CE and 87.7% of surgical MDs who agree that it is important to address diet in every visit. However, less than half of physicians regularly talk to their patients about maintaining a healthy weight or give them dietary recommendations [20]. Nutritional health requires effective, personalized, and motivating communication with patients, and primary care physicians should provide nutritional advice as well as basic nutrition recommendations as tools to promote good health [11, 20]. If doctors provided timely nutritional advice and referred patients to a nutritionist when necessary, the incidence of diet-associated diseases would decrease. ( [15, 21]).

**Table 3:** Adjusted associations between physician perception about nutrition and type of practice

Survey Item	Clinical		Surgical	
	Mean *±* SD	% Agree(*n*)	Mean *±* SD	% Agree(*n*)
**Beliefs around the value of nutrition**				
Nutrition assessment and counseling should be part of the routine care of all doctors, independent of their specialty.	4.6 *±* 0.7*	96.5(82)	4.3 *±* 1	89.8(44)
Inadequate nutritional advice can affect the result of medical treatment.	4.5 *±* 0.6	97.7(83)	4.6 *±* 0.5	100(49)
It is important to refer patients in whom a nutritional problem was identified to the qualified personnel.	4.8 *±* 0.4	100(85)	4.7 *±* 0.6	98(48)
It is important to advise patients about use of supplements and emphasize when they are contraindicated.	4.3 *±* 0.8*	91.8(78)*	3.8 *±* 1.3	77.6(38)
It is important to address the importance of the diet every time a patient is attended to.	4.6 *±* 0.6**	97.7 (83)*	4.4 *±* 1**	87.8(43)*
**Confidence in self and in patients**				
I feel comfortable with my capacity to talk about prevention strategies and nutrition treatment of illnesses such as osteoporosis, metabolic syndrome, hypertension, hyperthyroidism, stunting, gestational diabetes, and others.	3.6 *±* 1.2*	68.2(58)*	3.0 *±* 1.2	51 (25)
I feel capable of providing examples of the recommended food portions per the National or International guidelines	3.2 *±* 1.3	55.3(47)*	2.5 *±* 1.3*	32.7 (16)
I feel capable of recommending diet patterns for patients with chronic diseases	3.2 *±* 1.3***	52.9(45)**	2.3 *±* 1.1**	24.5 (12)*
I feel capable of recognizing nutritional risk in all of my patients.	3.6 *±* 1.1**	74.1(63)**	3.0 *±* 1.2	51.0 (25)
I believe that the majority of doctors are NOT adequately trained to discuss nutrition problems with patients.	4.1 *±* 0.8	89.4(76)	4.2 *±* 0.8	93.9(46)
Patients are NOT motivated to change unless they are sick.	3.8 *±* 1.1**	74.3(64)*	4.3 *±* 0.9	93.9(46)
**Personal knowledge about nutrition**				
I know the role that genetics, diet, and pharmacology play in weight loss regimens.	3.8 *±* 1.4	78.8(67)	3.6 *±* 1.2	73.5(36)
I know how to use and interpret BMI and waist circumference.	4.1 *±* 0.9**	89.3(75)*	3.6 *±* 1.1**	73.5(36)*
I know how many calories there are in one gram of fat, proteins, and carbohydrates, and their basic metabolic functions.	3.5 *±* 1.3*	67.9(57)*	2.9 *±* 1.4*	49 (24)*
I am well informed about the potentially harmful interactions between medications and herbal supplements or botanicals.	3.5 *±* 1.2***	65.9 (22)**	2.7 *±* 1.3*	40.8 (20)
I am well informed about the nutrient interactions of medications with food	3.3 *±* 1.2***	59.5(50)***	2.3 *±* 1.1***	26.5 (13)*
**Time allocation to address nutrition topics in practice**				
Doctors can have an effect on the eating behavior of a patient if they take the time to discuss the problem.	4.6 *±* 0.1	98.8(84)	4.6 *±* 0.5	100 (49)
It is NOT worth it to dedicate my time to advise patients with deficient eating patterns about nutrition.	1.5 *±* 0.7**	2.4 (2)**	2.1 *±* 1.2*	18.4 (9)*
Nutrition counseling is NOT an effective use of my time.	1.9 *±* 0.1***	9 (10.59)**	2.7*±*(1.3)	32.7 (16)

* *p* < 0.05, ** *p* < 0.01, *** *p* < 0.001

Second, it is important to explore the self-confidence of physicians in nutrition related issues. Our results demonstrated that 1 in 4 of CE do not feel capable of recognizing nutritional risk, and yet the Ecuadorian hospital malnutrition study, which included 5,355 patients in 36 public hospitals, found a prevalence of 37.2% of malnutrition in different medical hospitals [22]. Therefore, medical specialists are not screening for malnutrition, thus worsening the nutritional status of their patients [23]. Further, the diagnosis of over and undernutrition must become a priority among medical doctors, even more during the formative years of their education. In a survey about nutritional attitudes and routines among 4,512 Scandinavian health professionals, the discrepancy between standards and actual clinical practice to address hospital malnutrition was underscored. The most common cause of inadequate nutritional practice was insufficient knowledge followed by lack of interest and lack of responsibility [24].

Furthermore, our results indicated a significant difference between 24.5% of surgical MDs and 52.9% of CE who felt capable of recommending dietary patterns for patients with chronic diseases. This finding highlights the essentiality of physicians having an updated understanding of basic nutrition principles to best answer commonly asked questions. Physicians must also be aware of the role that each health professional has and the importance of nutrition both in the prevention and treatment of diseases, thus enabling the referral of patients to nutritionists to adhere to complementary treatment [11]. Nonetheless, general practitioners as well as specialists should all have a fundamental role in identifying food insecurity, preventing, and treating the double burden of malnutrition, as well as treating non-communicable chronic diseases associated with lifestyle [25]. According to data presented in this paper, only 55.3% of CE vs. 32.7% of surgical MDs feel capable of providing examples of the recommended food portions, another key element to the treatment and prevention of various conditions.

Third, Cardenas also suggested that despite knowing and being aware of the problem, neither the general practitioner nor the specialist physician are adequately trained or prepared to deal with the nutritional difficulties of the population [25]. In Ecuador, despite the well-known connection between chronic non-communicable diseases and poor diet, most universities include only one required nutrition course over 5–6 years of medical studies. Although the courses completed by medical students were often called ¨nutrition,” ¨nutrition and health,” ¨fundamentals of nutrition,” or ¨diet therapy,” the survey results demonstrate that perceptions around nutrition knowledge continue to vary among MDs. These results support the notion that insufficient nutritional knowledge among health professionals is the number one reason for inappropriate nutritional management on behalf of doctors [26]. Another study carried out in Ghana highlights medical students who believe their inadequate nutritional education is due to the low priority and importance given to the topic [15]. Despite consensus among health professionals and students that nutrition and dietetics have an adequate basis for clinical use in patient care, these disciplines remain scarce in education within medical schools [27].

It is important to note that, unlike the training of a medical student, nutritionists often undergo 4 years of undergraduate studies to receive a bachelor’s degree in nutrition and dietetics, and then continue on to complete at least 2 years of graduate education. These studies validate the nutrition professional’s ability to provide nutritional counseling services, as well as medical nutritional therapy to patients. Therefore, it is understandable that doctors indicate they lack the knowledge and skills to comply with nutritional standards in clinical practice [11].

In addition, one of the reasons doctors are not being ready to provide basic nutritional counseling for disease prevention and treatment is that the role they play in nutrition therapy lacks definition and prioritization [25]. In a research study conducted in the United States, it was concluded that inadequate nutrition education in medical schools continues to impact existing nutrition knowledge amongst MDs, as less than 25 h of nutrition education is required over a 4 year period of study [28]. To that point, researchers have contended that while courses such as organic chemistry have traditionally helped “weed out” applicants to medical school, a rigorous course in nutrition would integrate contemporary molecular biology, epidemiology, behavioral sciences, physiology, and additional important disciplines in which a medical doctor should be familiar with [29]. It is imperative for doctors to receive basic, yet robust, nutrition education so they have the ability to refer at-risk patients to trained specialists who can provide effective nutritional care. Such specialized care would complement prescribed medical treatment and bring about holistic changes in the patient’s eating behavior, which are further influenced by cultural, social and economic variables [11]. The lack of patients´ compliance of nutritional advice may be due to the limited time available for consultation and the insufficient skills of doctors to provide primary nutritional advice, as well as their inability to carry out multidisciplinary work together with nutritionists [30].

Fourth, as stated in the results section, almost 10% of clinical MD’s and 33% of surgical MD’s believe nutrition counseling is not an effective use of time. A very short consultation time has been described as one of the causes that prevents implementing good health promotion practices, including that of MDs providing nutritional advice [31]. There also exist some differences in the optimal length of medical consultation globally. In Spain it depends on the city, considering 20 min in Zaragoza and 10 min in Madrid. In Russia, the time allotted to consultation is 10 min, which are distributed as follows: 1 min to greet and say goodbye to the patient, 3 min for anamnesis, in 2 min a physical examination is performed, 2 min for the prescription and 2 min to carry out administrative duties. In the United States, the average consultation time increased from 16 to 20 min. Japan has the shortest time, being 6 min per query [32]. Even more, in Argentina the average medical attention time is fifteen minutes per patient, and it can increase in specialties such as pediatrics. In El Salvador, the doctor offers 10-minute appointments, while in Peru the appointments are 12 min [32].

This in mind, medical care times in Ecuador for adults should be approximately 20 min, while 30 min should be allotted for children and older adults [33]. While our study did not asses the average time spent with patients [32], determining how long the medical professional will be face to face with the patient is essential to organize the appointment schedule and also to guarantee the quality of medical care [31]. A decrease in medical consultation time reduces patient satisfaction and decreases medical prevention activities, which include giving basic nutritional counseling to patients or referring them to a nutritionist [31].

Continuing to look at trends across the globe, in Australia, nutrition education provided to patients with non-communicable diseases is rare. In addition, referral by doctors to a nutritionist when the patient is in need of specialized nutrition care is uncommon. This lack of importance around necessary referrals to a nutritionist often results in misinformation around nutrition topics among patients [34]. Several studies suggest that consultations should have a relative duration of 20 min to satisfy users and comply with quality care standards. In this way, the medical professional can focus on giving basic nutritional advice to their patients and direct them towards the nutrition specialist to accompany the treatment and obtain better results if needed [31]. Providing clear nutritional advice based on scientific evidence and awareness of the key role optimal, healthy eating habits play in the prevention and treatment of diseases, makes doctors crucial elements of a multidisciplinary network [11] Interdisciplinary work is needed between doctors who have the first contact with patients, and nutritionists [30]. This is the first study of its kind in Ecuador, serving as a starting point to understand the current situation of nutritional knowledge and education in MDs. It provides a steppingstone into curriculum modifications to include deeper nutritional training.

### Limitations

Some survey responses lacked complete data in the categories of age and graduation year. This required certain entries to be listed as “missing” in the Stata - version 18 software when conducting statistical analysis of sample characteristics. Moreover, our study did not explore how, if in any way, did beliefs, self-confidence, knowledge and time allocated to address nutrition concerns correlate, or, if place of work played a role in how survey items were answered, in addition to type of practice. Our survey did not inquire enough about primary place of work, (i.e. if most of the working week was spent working in a primary care facility or a hospital) to properly assess the influence such variables may have on survey items. Future studies should consider how to better investigate these queries. Information about referrals to nutrition professionals and actual nutritional management of health conditions was not collected.

## Conclusions

In Ecuador, while many MDs acknowledge the importance of nutrition in healthcare, a gap exists in their knowledge of basic nutrition. These findings align with previous studies which evidence the general lack of nutrition education among physicians on a global scale. Future advances should focus on developing professional models for nutrition education and training at the undergraduate and postgraduate levels among MDs, as well as creating standard nutrition guidelines for all medical personnel.

Moreover, integration of nutrition and dietetics into medical curricula should be prioritized to ensure physicians have a basic understanding of nutrition and its role in all health outcomes. The goal is for clinicians to acquire the knowledge and ability to provide basic nutrition advice and recognize when a referral is required. Incorporating these action steps into medical practice would effectively communicate to patients the importance of nutrition in their health [11].

It is essential to educate doctors and patients alike so that nutrition is accounted for as a key element of health care [11]. Effective collaboration between medical professionals and nutritionists can help achieve the changes in community behavior as it relates to knowledge, attitudes, and intended healthful habits. The lack of nutrition knowledge among doctors and the minimal importance given to the science of nutrition as a fundamental treatment has been recognized, and now that the problem has been identified, it is time to develop and implement strategies for change.

## Data Availability

No datasets were generated or analysed during the current study.
